# Medical Image Visual Appearance Improvement Using Bihistogram Bezier Curve Contrast Enhancement: Data from the Osteoarthritis Initiative

**DOI:** 10.1155/2014/294104

**Published:** 2014-05-20

**Authors:** Hong-Seng Gan, Tan Tian Swee, Ahmad Helmy Abdul Karim, Khairil Amir Sayuti, Mohammed Rafiq Abdul Kadir, Weng-Kit Tham, Liang-Xuan Wong, Kashif T. Chaudhary, Jalil Ali, Preecha P. Yupapin

**Affiliations:** ^1^Department of Biotechnology and Medical Engineering, Faculty of Biosciences and Medical Engineering, Universiti Teknologi Malaysia, 81310 Skudai, Johor, Malaysia; ^2^Department of Biotechnology and Medical Engineering, Medical Device and Technology Group, Material and Manufacturing Research Alliance, Faculty of Biosciences and Medical Engineering, Universiti Teknologi Malaysia, 81310 Skudai, Johor, Malaysia; ^3^Department of Radiology, School of Medical Sciences, Universiti Sains Malaysia, 16150 Kubang Kerian, Kelantan, Malaysia; ^4^Medical Device and Technology Group, Faculty of Biosciences and Medical Engineering, Universiti Teknologi Malaysia, 81310 Skudai, Johor, Malaysia; ^5^Department of Control Engineering and Mechatronic Engineering, Faculty of Electrical Engineering, Universiti Teknologi Malaysia, 81310 Skudai, Johor, Malaysia; ^6^Institute of Advanced Photonics Science, Nanotechnology Research Alliance, Universiti Teknologi Malaysia, 81310 Johor Bahru, Malaysia; ^7^Institute of Advanced Photonics Science, Nanotechnology Research Alliance, Universiti Teknologi Malaysia, 81310 Skudai, Johor, Malaysia; ^8^Department of Physics, Advanced Studies Center, Faculty of Science, King Mongkut's Institute of Technology Ladkrabang, Bangkok 10520, Thailand

## Abstract

Well-defined image can assist user to identify region of interest during segmentation. However, complex medical image is usually characterized by poor tissue contrast and low background luminance. The contrast improvement can lift image visual quality, but the fundamental contrast enhancement methods often overlook the sudden jump problem. In this work, the proposed bihistogram Bezier curve contrast enhancement introduces the concept of “adequate contrast enhancement” to overcome sudden jump problem in knee magnetic resonance image. Since every image produces its own intensity distribution, the adequate contrast enhancement checks on the image's maximum intensity distortion and uses intensity discrepancy reduction to generate Bezier transform curve. The proposed method improves tissue contrast and preserves pertinent knee features without compromising natural image appearance. Besides, statistical results from Fisher's Least Significant Difference test and the Duncan test have consistently indicated that the proposed method outperforms fundamental contrast enhancement methods to exalt image visual quality. As the study is limited to relatively small image database, future works will include a larger dataset with osteoarthritic images to assess the clinical effectiveness of the proposed method to facilitate the image inspection.

## 1. Introduction

Magnetic resonance (MR) imaging allows direct visualization of knee cartilage and quantitative measurement on cartilage to monitor osteoarthritis (OA) progression [1]. The advancement of MR imaging contributes greatly to the search for effective OA imaging biomarker [2]. Imaging biomarker is defined as “any anatomic, physiologic, biochemical, or molecular parameter detectable with one or more imaging methods used to diagnose the presence and/or severity of disease [3].” Since imaging biomarker is crucial for developing disease modifying osteoarthritis drug (DMOAD) [4], the National Institutions of Health (NIH) has launched the Osteoarthritis Initiative (OAI) in a public-private consortium. To date, a total of 4,796 participants have been recruited from four clinical institutions (University of Maryland, School of Medicine, Baltimore, Md; Ohio State University, Columbus, OH; University of Pittsburgh, Pittsburgh, Pa; Memorial Hospital of Rhode Island, Pawtucket, RI) to provide comprehensive analysis for OA progression and identification of suitable OA biomarkers.

There have been growing interests in interactive segmentation recently [5–8]. Medical images such as knee MR images are usually too complex for fully automated segmentation to produce satisfactory results. On the other hand, interactive segmentation reflects user intention because the segmentation is initiated according to minimal user intervention in the forms of scribbles [9] or bounding box [10]. Therefore, unnecessary segmentation error such as oversegmentation reported in automated segmentation [11] can be avoided. Typically, interactive segmentation can be divided into two groups: boundary based and contour based. Although both groups present different segmentation approaches, initialization of these methods will greatly depend on human knowledge. Therefore, direct incorporation of human knowledge to commence the segmentation translates into underlying need for human interpretation of MR image. Distinct feature manifestation and excellent visual quality of the medical image, for instance, play important role to assist user in interpreting medical images and then inserting seeds onto region of interests.

The visual quality of knee MR image can be deteriorated by several phenomena: insignificant tissue contrast, complex knee joint structure, and low background luminance. Definitive tissue contrast facilitates the image interpretation process. Unfortunately, mediocre tissue contrast has been observed in knee MR image. For example, tissue contrast between cartilage, surrounding muscle tissues, and image background only differ slightly. As a result, poor contrast difference observed in the image can easily lead to inter- and intraobserver ambiguity during inspection. Besides, human knee structure is anatomically complex. The knee compartment is filled with various types of cartilages, knee bones, muscle, fat tissue, and ligaments; attempting to interpret MR image equipped with poor tissue contrast is laborious. Further, MR image of knee is characterized by large numbers of low intensity background pixels. Dark background hardens any effective identification of pertinent image features. Eventually, above mentioned phenomena give rise to the prominence of a tissue contrast enhancement model to elevate image visual quality.

Contrast enhancement is defined as a remapping process to transform the image's intensity distribution so the image intensity range can be fully exploited [12]. In particular, contrast enhancement has been implemented to extract salient information, protrude image features, and improve the image's visual quality. Applications of various contrast enhancement models on medical images for further analysis have been reported in previous studies. Ismail and Sim [13] applied dynamic contrast enhancement on low resolution brain MR images, so important brain image features could be protruded. Chai et al. [14] proposed the use of multipurpose contrast enhancement technique to overcome the poor contrast problem reported in the ossification sites of children hand bones. In the context of knee MR image, adequate contrast enhancement to ameliorate the image appearance will be meaningful enough for the subsequent interactive knee cartilage segmentation to succeed.

Direct contrast enhancement through traditional histogram equalization [12] cannot be implemented into medical image processing. Traditional histogram equalization distorts the brightness enhancement by invariably shifting output mean brightness to middle gray level. The resultant image, therefore, is over-enhanced and any brightness sensitive image feature is destroyed. Besides, traditional histogram equalization combines any neighboring gray levels with light probabilistic density into one gray level, widening the gap with neighboring gray levels which have heavy probabilistic density. The flawed intensity redistribution contributes to severe detail image loss and sudden jump in cumulative density function.

Fundamental contrast enhancement betterments are developed based on the idea of histogram partitioning. The gist of these improved models concentrates on preserving mean brightness of original image using different intensity threshold values, that is, mean and median. As such, one of the earliest bihistogram equalization methods, brightness preserving bihistogram equalization (BBHE) [15] has been developed based on this concept. BBHE partitions the global histogram into two subhistograms according to mean intensity value. Then traditional histogram equalization is implemented independently onto the subhistograms. Given that BBHE partitions the intensity distribution into two, while mean brightness of input image is preserved. Conservation of mean brightness is deemed imperative in preventing resultant image from severe intensity distortion.

In 1999, Wang et al. [16] introduced dualistic subimage histogram equalization (DSIHE). This method uses median intensity value to decompose original image's histogram. Yu postulates that maximum segmentation entropy can be obtained when two subimages have equal areas. DSIHE is capable of preserving original image's mean brightness as well as attaining the most image entropy. Meanwhile, minimum mean brightness error bihistogram equalization (MMBEBHE) [17] has developed based on iterative selection of optimal threshold value. For instance, the optimal threshold value must satisfy minimum absolute mean brightness error (AMBE) between output histogram and input histogram. Hence, MMBEBHE overcomes the problem of unnatural enhancement and maximizes the degree of brightness preservation; consequently, it downplays the creation of unwanted artifacts. However, MMBEBHE is relatively unsuitable in real image processing because generating AMBE for each possible input intensity level is computationally complicated.

Chen and Ramli [18] proposed an iterative extension of BBHE known as recursive mean separate histogram equalization (RMSHE). RMSHE decomposes original image histogram into several subimages based on mean value and performed traditional histogram equalization up to *r* times. RMSHE claims to demonstrate better brightness preservation when the number of iterations increases. Instead, higher numbers of iterations reduce the enhancement effect to produce highly identical output image. The iterative extension of DSIHE, recursive subimage histogram equalization (RSIHE), has been developed by Sim et al. [19]. Unfortunately, RSIHE shares similar problem with RMSHE. Furthermore, both recursive HE models have computational power up to  2^*r*^. If higher degree of histogram decomposition is chosen, the process would become computationally complicated.

In this paper, a new contrast enhancement model known as bihistogram Bezier curve contrast enhancement (BBCCE) is proposed. The intention of BBCCE is to improve image visual quality through appropriate contrast enhancement without compromising the conservation of salient knee features. As such, transformation of intensity distribution is performed using remapping process based on Bezier transform curve instead of conventional cumulative density function. Besides, the important property of bihistogram equalization is retained by partitioning the histogram into two subhistograms to curtail predominance of low intensity background pixels.

## 2. Materials and Methods

### 2.1. MR Image Acquisition

Twenty healthy baseline (Data set: 0.C.2) dual-echo steady-state (DESS) knee MR images with water excitation (we) [20] from the OAI dataset are selected. MR images are acquired using 3.0 T MRI scanner (Siemens Magnetom Trio, Erlangen, Germany) with quadrature transmit-receive knee coil (USA Instruments, Aurora, OH). In this experiment, the DESSwe MR images have section thickness of 0.7 mm and in-plane resolution of 0.365 × 0.456 mm. Other technical parameters are outlined as follows: field of view = 140 × 140 mm, flip angle = 25°, TR/TE = 16.3/4.7 m sec, matrix size = 384 × 384 mm, bandwidth = 185 Hz/pixel. Further information related to the OAI dataset could be found through: http://oai.epi-ucsf.org/datarelease/About.asp.

### 2.2. Contrast Enhancement Model Overview

**Figure 1:**
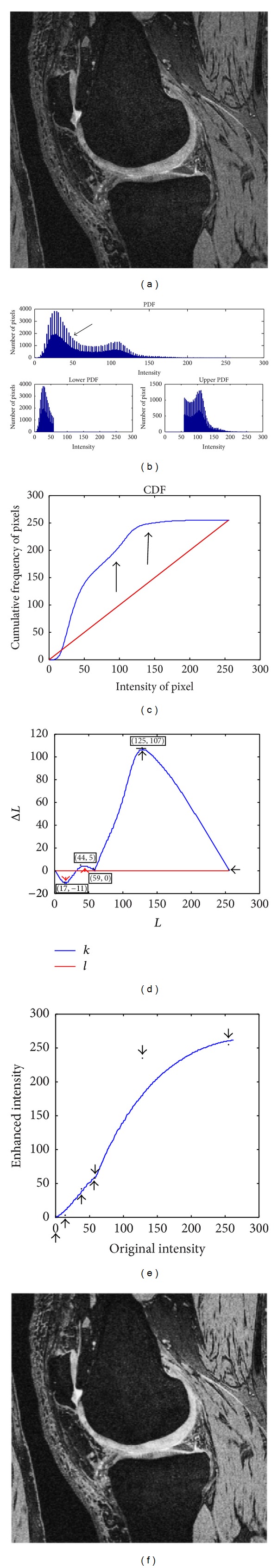
Flow of BBCCE computation using knee MR image. (a) Original MR image. (b) Upper diagram shows histogram of original MR image where black arrow indicates dominant background intensities and lower diagrams show the decomposition of histogram into lower and upper subhistograms in BBCCE. (c) Cumulative density function of original MR image where black arrows indicate large intensity distortion which contributes to sudden jump issue. (d) Intensity discrepency curve duduced from cumulative density function where upward and downward red arrows indicate global maximum and global minimum in lower histogram while upward black arrow indicates global maximum in upper histogram. Leftward black arrow defines the boundary for global extremum in intensity discrepency curve. (e) Bezier transform curve generated using control points duduced from intensity discrepency curve. (f) BBCCE enhanced MR image.

Conventional transform curve is derived from cumulative density function. The objective is to stretch the original intensity distribution using transform curve to cover full dynamic range of the image. Thus, image's contrast can be modified. However, cumulative density function based remapping process does not always yield the desired effect because distortion by dominant intensity levels (as indicated by arrow in Figure 1(b)) could easily induce sudden jump in conventional transform curve (as indicated by arrows in Figure 1(c)), resulting in abrupt rise of transformed intensity values. On the ground of above mentioned concerns, it is believed that an adequate tissue contrast improvement is most appropriate approach for DESSwe knee MR image.

The term “adequate” implies dynamic adjustment of transformed intensity values to refine the traditional transform curve. In addition, we must preserve prominent image features and maintain natural visual appearance simultaneously. Hence, the proposed BBCCE uses the largest intensity discrepancy value deduced from intensity difference curve (Figure 1(d)) to identify furthest intensity distortion caused by traditional histogram equalization. This model, therefore, could handle arbitrary intensity variability exhibited by different images. Then, global extrema/extremum is computed from the curve and used as control points to generate Bezier transform curves. Finally, the Bezier curves are concatenated to form global transform curve (Figure 1(e)). BBCCE is developed by using MATLAB (Mathworks, Natick, MA).

### 2.3. Image Decomposition

MR image is defined with *L* discrete gray levels as *X* = {*X*(*i*, *j*)} where *X*_*k*_(*i*, *j*) ∈ {*X*_*o*_, *X*_1_,…, *X*_*L*−1_}.   By treating these pixel intensities as random variables, the probability density function is estimated as, *p*(*X*_*k*_) for MR image. Probability density function depicts how frequent certain pixel intensity occurs. Hence, probability density is generated by dividing the pixel occurrence of certain gray level *k*, *n*_*k*_ over the total number of pixels, *n*. Consider the following:

(1) $$\begin{array}{c} p\left(X_{k}\right)=\frac{n_{k}}{n}. \end{array}$$

In this work, two subhistograms are produced after histogram decomposition. The partition is intended to confine any abrupt distribution skew caused by large amounts of background pixels to lower histogram. To compute histogram decomposition, the mean MR image pixel intensity *μ* is calculated. Formulation for the arithmetic mean is illustrated as follows:

(2) $$\begin{array}{c} \mu =\frac{\sum_{k=0}^{k}n_{k}\left(X_{k}\right)}{n}=\overset{k}{\underset{k=0}{\sum }}p\left(X_{k}\right)X_{k}. \end{array}$$

### 2.4. Intensity Difference Curve

Histogram of MR image can be derived from the plot of *n*_*k*_ against  *X*_*k*_. To attain contrast enhancement through histogram equalization, basic idea is to stretch the input histogram to resemble uniform distribution regardless of the initial histogram shape [12]. However, pixel intensity is presented in discrete domain for real image processing, and the output probability density function could only get as close as possible to the uniform distribution. During the implementation of histogram equalization, cumulative density function plays nontrivial role to remap the pixel intensity distribution. The conventional cumulative density function as *c*(*x*) and traditional transform function as *f*(*x*) can be defined as

(3) $$\begin{array}{c} c\left(x\right)=\overset{k}{\underset{k=o}{\sum }}p\left(X_{k}\right),\\ f\left(x\right)=X_{o}+\left(X_{L-1}-X_{o}\right)c\left(x\right). \end{array}$$

As shown in (3), traditional transform function relies heavily on conventional cumulative density function without considering the sudden jump issue. Sudden jump in conventional cumulative density function arises when predominate pixel intensity instigates steep increase in the function. Consequently, steep increment over small intensity range induces excessive brightness lift. This issue requires serious attention in the case of knee MR image since large amount of background pixels can skew conventional cumulative density function.

In proposed method, the solution for this issue is to smooth the conventional cumulative density function based on intensity distortion caused by sudden jump. Noteworthy, the degree of contrast enhancement depends on absolute intensity difference (AID), which is defined as

(4) $$\begin{array}{c} \text{AID}=x^{\prime }-x, \end{array}$$

where *x*′ indicates the transformed intensity and *x* is the original intensity. The degree of contrast enhancement could be decreased by lowering the AID.

The intensity difference curves are deduced by computing discrete AIDs for both subhistograms. Notably, intensity difference curve for each MR image is distinct. This novel feature in the proposed BBCCE allows us to consider arbitrary intensity distribution variation for smoothing conventional cumulative density function. Thus, the transform curve is based on two factors: input intensity distribution and resultant intensity fluctuation. Suppose that the intensity difference curve is defined as *y* = *I*(*x*). Then, intensity discrepancy value, IDV ∈ *R*. IDV illustrates the intensity discrepancy for intensity level that corresponds to the critical point where its gradient is equal to zero, *dy*/*dx*  = 0. Within the predefined range, several critical points could exist potentially. These critical points, *P*(*x*) as local minimum, can be identified if *d* ^2^ *y*/*dx* ^2^ > 0 and local maximum if  *d* ^2^ *y*/*dx* ^2^ < 0. The critical points are subjected to self-imposed boundary condition *y* = 0 where local minimum is only defined in region *y* < 0 and local maximum is defined in region  *y* > 0. Thus, global extrema could be found among these critical points by searching for the largest IDV under predefined constraints.

(5) $$\begin{array}{c} P_{\text{Global}\;\text{maximum}}\left(x\right)=\{\begin{array}{cc} \text{Argmax}\left(\text{IDV}\right); & y>0\\ 0; & \text{otherwise}, \end{array}\\ P_{\text{Global}\;\text{minimum}}\left(x\right)=\{\begin{array}{cc} \text{Argmin}\left(-\text{IDV}\right); & y<0\\ 0; & \text{otherwise}. \end{array} \end{array}$$

Given that the proposed method defines MR image within [0, *L* − 1], first search attempt is performed in the range of 0 to *μ* (lower histogram) and *μ* to *L* − 1 (upper histogram) for second search attempt. Eventually, two sets of global extremum or global extrema are obtained.

### 2.5. Bezier Transform Curve

Bezier curve is popular in computer graphics and computer-aided application. The parametric curve, representing the special case of *B*-spline, has been instrumental for widely diversified applications spanning from industrial shape design to game development. Inherently, Bezier curve is characterized by convex hull. The curve is contained inside control polygon, guaranteeing that the generated curve will not derail off its control polygon. In spite of its versatility and advantages, to our best knowledge, multiple Bezier curves have never been applied in medical image processing. Here in after, Bezier curves are introduced to produce smooth remap curve that yields smaller AID. Denote the control points as *P*_0_, *P*_1_,…, *P*_*n*_ and is bounded within the domain *t* ∈ [0,1], Bezier curve can be define as [21]

(6) $$\begin{array}{c} Q\left(t\right)=\overset{n}{\underset{i=0}{\sum }}B_{n,i}\left(t\right)P_{i}. \end{array}$$

Bezier curve uses Bernstein polynomial *B*_*n*,*i*_(*t*) as its basis function, in which definition of the polynomial is formulated in (7). Consider the following:

(7) $$\begin{array}{c} B_{n,i}\left(t\right)=\left(\begin{array}{c} n\\ i \end{array}\right)t^{i}{\left(1-t\right)}^{n-i}, \end{array}$$

where the binomial coefficient $\left(\begin{array}{c} n\\ i \end{array}\right)$ is given as

(8) $$\begin{array}{c} \left(\begin{array}{c} n\\ i \end{array}\right)=\{\begin{array}{cc} \frac{n!}{i!\left(n-1\right)!} & \text{if}\;0\leq i\leq n\\ 0 & \text{otherwise}. \end{array} \end{array}$$

Based on intensity difference curve, Bezier curve of second degree or third degree is likely to be generated. If a pair of global extrema is detected from the curve, Bezier curve of third degree, *n* = 3 can be deduced with four control points  *P*_*i*_ ∈ {*P*_0_, *P*_1_, *P*_2_, *P*_3_}. On the other hand, Bezier curve of second degree, *n* = 2 with three control points *P*_*i*_ ∈ {*P*_0_, *P*_1_, *P*_2_} is selected if global extremum is detected. The point selection is automated in our method. Specific second and third degree Bezier curves are expressed as follows:

(9) $$\begin{array}{c} Q\left(t\right)=\{\begin{array}{cc} \overset{2}{\underset{i=0}{\sum }}\left(\begin{array}{c} 2\\ i \end{array}\right)t^{i}{\left(1-t\right)}^{2-i}P_{i}; & n=2\\ \overset{3}{\underset{i=0}{\sum }}\left(\begin{array}{c} 3\\ i \end{array}\right)t^{i}{\left(1-t\right)}^{3-i}P_{i}; & n=3. \end{array} \end{array}$$

### 2.6. Assessment Methodology

The qualitative assessment and statistical analysis is conducted to evaluate the performance of traditional histogram equalization (referred to THE in Section 3), BBHE, DSIHE, RMSHE (*r* = 2), RSIHE (*r* = 2), and BBCCE. Image visual quality is conducted by assessing the effect of improved tissue contrast on structural delineation relative to original image. The radiologists are consulted regarding the tissue contrast improvement effect produced by different types of methods. The performance of various contrast enhancement methods from statistical perspective in terms of contrast enhancement degree (EME), intensity distortion (AMBE), and image quality assessment (FSIM) is also examined. The purpose of statistical analysis is to test the hypothesis that BBCCE outperforms fundamental contrast enhancement methods.

Given an original image, *X* and its resultant image  *R* measure of enhancement (EME) gauges the image enhancement of resultant image [22]. Using a block size of 3 × 3 for each of the nonoverlapping blocks  *b*_1_ *b*_2_, EME estimates the average contrast in every block by averaging the maximum intensity value, *I*_max⁡_ and minimum intensity value,  *I*_min⁡_. The image contrast is enhanced if EME of image *R* is greater than image  *X*. In this experiment, it is believed that the lesser EME values (but greater than original EME value) would be adequate to improve tissue contrast in knee MR image. Consider the following:

(10) $$\begin{array}{c} \text{EME}=\underset{\Phi \in \{\Phi \}}{\max}\chi \left(\frac{1}{b_{1}b_{2}}\overset{b_{2}}{\underset{l=1\;}{\sum }}\overset{b_{1}}{\underset{k=1}{\sum }}20\log\frac{I_{\max}}{I_{\min}}\right), \end{array}$$

where {Φ} indicates orthogonal transforms and *χ*(*x*) is the sign function.

Mean brightness difference between original image and resultant image reveals the degree of luminance distortion. The statistical evaluation metric, known as absolute mean brightness error (AMBE), is defined as

(11) $$\begin{array}{c} \text{AMBE}\left(R,X\right)=\left|E\left(R\right)-E\left(X\right)\right|, \end{array}$$

where *E*(·) denotes the expectation, *R* represents the resultant image, and *X* represents the original image. A lower AMBE is translated into better brightness preservation. The AMBE are normalized and twenty MR images have been included in experiment. The formulation for average AMBE is shown in (12). For instance, smaller AMBE implies lesser mean intensity distortion (lesser image information loss). AMBE value (AMBE = 0) is taken as reference from original MR image.

(12) $$\begin{array}{c} \text{Average}\;\text{AMBE}=\frac{1}{N}\overset{N}{\underset{i=1}{\sum }}\text{AMBE}\left(R_{i},X_{i}\right). \end{array}$$

Feature similarity index model (FSIM) aims to perform image quality assessment (IQA) [23] for THE, BBHE, DSIHE, RMSHE, RSIHE, and BBCCE. FSIM has the maximum score of one; indicating the highest enhanced image quality. Suppose that two images *f*_1_ and *f*_2_ are included to calculate the similarity between these two images, then FSIM is defined as follows:

(13) $$\begin{array}{c} \text{FSIM}=\frac{\sum_{X\in \Omega }S_{L}\left(x\right)\cdot {\text{PC}}_{m}\left(x\right)}{\sum_{X\in \Omega }{\text{PC}}_{m}\left(x\right)}, \end{array}$$

where PC_*m*_(*x*) is the dimensionless feature perceived at a point where the Fourier components reach maximal in phase, *S*_*L*_(*x*) is the overall similarity between images, and *f*_1_ and *f*_2_ and *Ω* are the whole image spatial domain.

## 3. Results and Discussion

### 3.1. Qualitative Results

**Figure 2:**
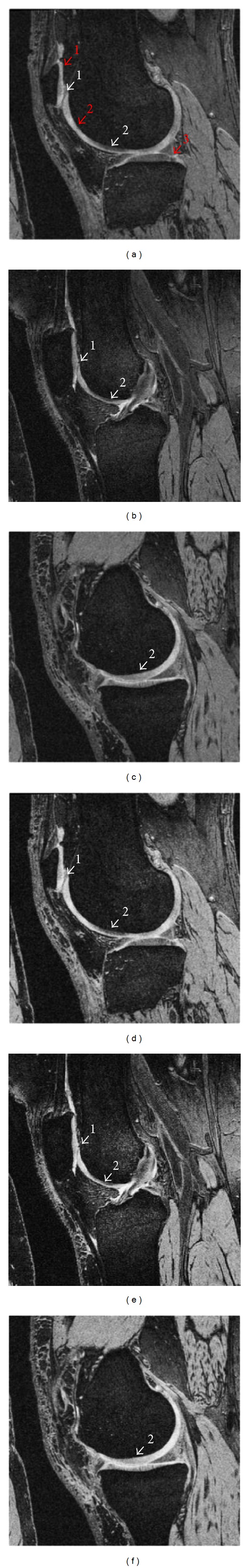
Manifestations of contrast enhancement effect on MR image from medial, central, and lateral sides of the knee joint to represent overall enhancement impact on the stack of 2D MR images. (First row from left to right) Original MR knee image from different sides: (a) medial, (b) central, and (c) lateral. (Second row from left to right) BBCCE enhanced MR knee image from different sides: (d) medial, (e) central, and (f) lateral. Knee cartilage is known as (in red arrows with label): (1) patellar cartilage, (2) femoral cartilage, (3) tibial cartilage. Prominent knee features in this image include (in white labeled arrows): (1) femoral sulcus and (2) Intensity variation within cartilage.

We considered several factors when assessing visual quality of BBCCE enhanced image such as natural looking, tissue contrast improvement, preservation of knee features, and minimum image artifact provocation. Effect of BBCCE enhancement is illustrated in Figure 2. As such, BBCCE improves tissue contrast (red arrows with labels: (1) patellar cartilage, (2) articular cartilage, and (3) meniscal cartilage) without obliterating knee features (white arrows with labels: (1) femoral sulcus and (2) slight intensity variation within the cartilage). Thus, cartilage becomes more differentiable from its surrounding tissues and bones. Besides, not much image noise has been amplified after contrast enhancement. Thus, well defined cartilage delineation, appropriate contrast enhancement, preservation of pertinent cartilage features, and minimal noise amplification produce natural appearance for the resultant MR image of knee.

**Figure 3:**
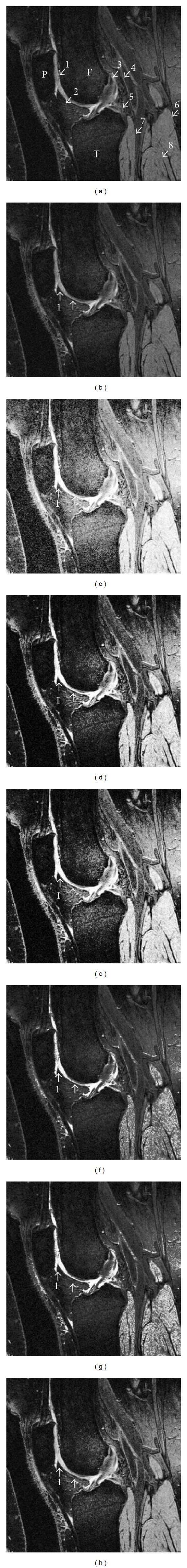
Central region manifestation of the left patellofemoral joint section using DESSwe MR imaging sequence. Labels in (a) indicate various skeletal elements inside knee joint (P = patellar, T = tibia, F = femur; 1 = femoral sulcus, 2 = Hoffa fat pad, 3 = anterior cruciate ligament (ACL), 4 = oblique popliteus ligament, 5 = posterior cruciate ligament (PCL), 6 = gastrocnemius muscle, 7 = popliteus muscle, 8 = soleus muscle). For comparison purpose, original MR image is remanifested in (b). Enhanced MR images using (c) THE, (d) BBHE, (e) DSIHE, (f) RMSHE, (g) RSIHE, and (h) BBCCE show the contrast enhancement effect relative to (b). The femoral sulcus (white arrow labeled as “1”) and intensity variation within cartilage (unlabeled white arrow) are indicated in images from (b) to (h).

Besides, BBCCE is compared to fundamental contrast enhancement techniques with relative to original MR image. Knee joint is a compound joint packed with various skeletal elements. Interpretation of complex MR image could lead to human ambiguity as a result of unclear structural delineation and unpleasant background image illustration.  Figure 3 demonstrates various knee components observed from MR image and the enhancement effect imposed by different contrast enhancement methods.

Typically, all contrast enhancement methods improve the tissue contrast, lifting the image's background luminance. However, apparent image noise amplification is detected in some previous contrast enhancement methods. For example, image noise amplified by traditional histogram equalization (THE) is obvious especially at femur and tibia. Irritating image artifact downgrades the visual quality. Besides, serious noise amplification is detected at popliteus muscle, gastrocnemius muscle, and soleus muscle in images produced by RSIHE and RMSHE. Thus, THE, RSIHE, and RMSHE are largely unsuitable for medical image contrast enhancement.

Preservation of salient information describing knee cartilage is imperative. Precise structural delineation through adequate tissue contrast refinement can maintain small but important feature details. For instance, BBCCE improves the cartilage contrast as well as conserves paramount details like femoral sulcus (white arrow labeled as “1”) and intensity variation within cartilage (unlabeled white arrow) simultaneously. Previous contrast enhancement methods, on the other hand, over-enhance the cartilage. Hence, intensity variation is destroyed and patellar cartilage is seen to “combine” with femoral cartilage.

### 3.2. Statistical Analysis

All tests are performed using SPSS (version 21). In this study, the hypothesis that BBCCE could outperform other contrast enhancement methods is tested and verified.  Table 1 shows the mean values for EME, FSIM, and AMBE of different contrast enhancement methods (THE, BBHE, DSIHE, RMSHE, RSIHE, BBCCE) using 20 healthy subjects. The results are evaluated by taking original image values (EME = 32.28; AMBE = 0; FSIM = 0-1) as reference. EME assesses the performance of contrast enhancement methods in terms of enhancement degree. It is deduced that BBCCE (41.44 ± 1.06) produces the least enhancement followed by RMSHE (43.33 ± 1.80). AMBE assesses the performance of contrast enhancement methods from intensity distortion perspective. RMSHE (14.02 ± 1.29) is ranked first in AMBE test and is followed closely by BBCCE (14.82 ± 1.47). FSIM assesses the performance of contrast enhancement methods from quality of image perspective. For instance, BBCCE (0.92 ± 0.02) is ranked first in producing highest FSIM score followed by RMSHE (0.83 ± 0.03). It is observed that BBCCE and RMSHE produces almost similar results and further analysis should be performed to test our hypothesis.

Table 2 indicates the contrast enhancement methods which impose significantly different impact on the MR images in all cases, that is, EME, AMBE, and FSIM (*P* < 0.05). Therefore, the data is further analyzed using post hoc tests (Fisher's Least Significant Difference and the Duncan test) to evaluate the performance of different contrast enhancement methods.

Fisher's least significant difference test (Table 3) indicates the mean differences between THE and DSIHE (0.61; −0.61 for DSIHE and THE) which is insignificant in EME. Other contrast enhancement methods show significant mean difference. The result is confirmed by the Duncan test (Table 4), which categorized THE and DSIHE into the same subset.

Fisher's least significant difference test (Table 5) indicates the mean differences between RMSHE and BBCCE (−0.79; 0.79 for BBCCE and RMSHE) which is insignificant in AMBE. Other contrast enhancement methods show significant mean difference. The result is confirmed by the Duncan test (Table 6), which categorized RMSHE and BBCCE in the same subset.

Fisher's least significant difference test (Table 7) indicates the mean differences between RMSHE and RSIHE (−0.01; 0.01 for RSIHE and RMSHE) which is insignificant in FSIM. Other contrast enhancement methods show significant mean difference. The result is confirmed by the Duncan test (Table 8), which categorized RMSHE and RSIHE in the same subset.

The performance of contrast enhancement methods (THE, BBHE, DSIHE, RMSHE, RSIHE, and BBCCE) is ranked according to the results computed from EME, AMBE, and FSIM in Table 9. Empty spaces that are observed from Table 9 show the readjustment made after conducting post hoc tests. For instance, it is found that BBCCE is outperformed consistently in all experiments. BBCCE (bolded) is ranked first in EME and FSIM while RMSHE is ranked equally with BBCCE in AMBE. Besides, it is concluded that THE consistently occupies at near bottom of the ranking, indicating the severe limitations faced by conventional histogram equalization to improve medical image contrast.

## 4. Conclusion

In this paper, bihistogram Bezier curve contrast enhancement (BBCCE) is presented. The objective of BBCCE is to assist radiologist to interpret MR image prior to performing knee cartilage segmentation. However, MR image possesses poor tissue contrast, and conventional contrast enhancement methods have failed to address this issue. As such, sudden jump in conventional transform curve causes the image to be over-enhanced, and deteriorates the image visual quality. Therefore, it is believed that the adequate contrast enhancement is the most appropriate solution as in the case of MR image. To achieve the objective, the Bezier transform curve based on intensity discrepancy value and intensity difference curve is deduced. The quantitative results show that BBCCE excels in terms of tissue improvement, minimal mean intensity distortion, and image quality. The results are in-line with qualitative results which show that BBCCE could preserve important knee features and better delineate the knee structure without provoking much image noise. In the future, the mean opinion survey and record image evaluation time to assess the effectiveness of BBCCE will be performed in assisting radiologists to interpret knee MR image.

## Figures and Tables

**Table 1:** Mean EME, AMBE, and FSIM values computed from THE, BBHE, DSIHE, RMSHE, RSIHE, and BBCCE by taking original image's EME (32.38) and AMBE (0.00) as reference. FSIM ranged from 0 to 1, where 1 indicates the best enhanced image quality.

Methods	Mean EME ± SD	95% confidence interval of the difference		Mean AMBE ± SD	95% confidence interval of the difference		Mean FSIM ± SD	95% confidence interval of the difference	
		Lower	Upper		Lower	Upper		Lower	Upper
THE	47.62 ± 2.05	46.66	48.58	77.39 ± 4.99	75.05	79.73	0.72 ± 0.02	0.71	0.74
BBHE	45.70 ± 1.92	44.79	46.60	27.35 ± 2.61	26.13	28.57	0.79 ± 0.03	0.78	0.81
DSIHE	47.01 ± 1.81	46.16	47.86	35.80 ± 2.34	34.71	36.90	0.77 ± 0.03	0.76	0.79
RMSHE	43.33 ± 1.80	42.48	44.17	14.0 ± 1.29	13.42	14.63	0.83 ± 0.03	0.82	0.85
RSIHE	54.74 ± 1.88	53.86	55.62	17.40 ± 1.19	16.84	17.96	0.82 ± 0.03	0.81	0.83
BBCCE	41.44 ± 1.06	40.94	41.93	14.82 ± 1.47	14.13	15.51	0.92 ± 0.02	0.91	0.93

**Table 2:** The one-way ANOVA computed by using different contrast enhancement methods in EME, AMBE, and FSIM.

	Sum of squares	df	Mean square	*F*	*P* value
EME					
Methods	2111.80	5	422.36	132.72	0.00*
Errors	362.80	114	3.18		
Total	**2474.60**	**119**			
FSIM					
Methods	58467.66	5	11693.53	1652.39	0.00*
Errors	806.75	114	7.08		
Total	**59274.40**	**119**			
AMBE					
Methods	0.42	5	0.09	124.12	0.00*
Errors	0.08	114	0.00		
Total	**0.50**	**119**			

*Significant *P* value (*P* < 0.05).

**Table 3:** Categorization of different methods using Fisher's Least Significance Difference (LSD) for EME.

(*I*) Method	(*J*) Method	Mean difference (*I*−*J*)	Std. error	*P* value	95% confidence interval	
					Lower bound	Upper bound
THE	BBHE	1.92*	0.56	0.00	0.81	3.04
	DSIHE	0.61	0.56	0.28	−0.51	1.73
	RMSHE	4.29*	0.56	0.00	3.17	5.41
	RSIHE	−7.12*	0.56	0.00	−8.23	−6.00
	BBCCE	6.18*	0.56	0.00	5.07	7.30

BBHE	THE	−1.92*	0.56	0.00	−3.04	−0.81
	DSIHE	−1.31*	0.56	0.02	−2.43	−0.20
	RMSHE	2.37*	0.56	0.00	1.25	3.48
	RSIHE	−9.04*	0.56	0.00	−10.16	−7.92
	BBCCE	4.26*	0.56	0.00	3.14	5.38

DSIHE	THE	−0.61	0.56	0.28	−1.73	0.51
	BBHE	1.31*	0.56	0.02	0.20	2.43
	RMSHE	3.68*	0.56	0.00	2.56	4.80
	RSIHE	−7.73*	0.56	0.00	−8.84	−6.61
	BBCCE	5.57*	0.56	0.00	4.46	6.70

RMSHE	THE	−4.29*	0.56	0.00	−5.41	−3.17
	BBHE	−2.37*	0.56	0.00	−3.48	−1.25
	DSIHE	−3.68*	0.56	0.00	−4.80	−2.56
	RSIHE	−11.41*	0.56	0.00	−12.53	−10.29
	BBCCE	1.89*	0.56	0.00	0.78	3.01

RSIHE	THE	7.12*	0.56	0.00	6.00	8.23
	BBHE	9.04*	0.56	0.00	7.92	10.16
	DSIHE	7.73*	0.56	0.00	6.61	8.84
	RMSHE	11.41*	0.56	0.00	10.29	12.53
	BBCCE	13.30*	0.56	0.00	12.18	14.41

BBCCE	THE	−6.18*	0.56	0.00	−7.30	−5.07
	BBHE	−4.26*	0.56	0.00	−5.38	−3.14
	DSIHE	−5.57*	0.56	0.00	−6.69	−4.46
	RMSHE	−1.89*	0.56	0.00	−3.01	−0.78
	RSIHE	−13.30*	0.56	0.00	−14.42	−12.18

*The mean difference is significant at the 0.05 level.

**Table 4:** Categorization of contrast enhancement methods into homogenous subset using the Duncan test for EME.

Method	*N*	Subset for alpha = 0.05				
		1	2	3	4	5
BBCCE	20	41.44				
RMSHE	20		43.33			
BBHE	20			45.70		
DSIHE	20				47.01	
THE	20				47.62	
RSIHE	20					54.74
Sig.		1.00	1.00	1.00	0.28	1.00

Means for groups in homogeneous subsets are displayed.

Harmonic Mean Sample Size = 20.000.

**Table 5:** Categorization of different methods using Fisher's Least Significance Difference (LSD) for AMBE.

(*I*) Method	(*J*) Method	Mean difference (*I* − *J*)	Std. error	*P* value	95% confidence interval	
					Lower bound	Upper bound
THE	BBHE	50.04*	0.84	0.00	48.38	51.71
	DSIHE	41.59*	0.84	0.00	39.92	43.25
	RMSHE	63.37*	0.84	0.00	61.70	65.03
	RSIHE	60.00*	0.84	0.00	58.32	61.66
	BBCCE	62.57*	0.84	0.00	60.91	64.24

BBHE	THE	−50.04*	0.84	0.00	−51.71	−48.38
	DSIHE	−8.45*	0.84	0.00	−10.12	−6.79
	RMSHE	13.33*	0.84	0.00	11.66	14.99
	RSIHE	9.95*	0.84	0.00	8.28	11.62
	BBCCE	12.53*	0.84	0.00	10.86	14.20

DSIHE	THE	−41.59*	0.84	0.00	−43.25	−39.92
	BBHE	8.45*	0.84	0.00	6.79	10.12
	RMSHE	21.78*	0.84	0.00	20.11	23.45
	RSIHE	18.40*	0.84	0.00	16.74	20.07
	BBCCE	20.98*	0.84	0.00	19.32	22.65

RMSHE	THE	−63.37*	0.84	0.00	−65.03	−61.70
	BBHE	−13.33*	0.84	0.00	−15.00	−11.66
	DSIHE	−21.78*	0.84	0.00	−23.45	−20.11
	RSIHE	−3.38*	0.84	0.00	−5.04	−1.71
	BBCCE	−0.79	0.84	0.35	−2.46	0.87

RSIHE	THE	−60.00*	0.84	0.00	−61.66	−58.32
	BBHE	−9.95*	0.84	0.00	−11.62	−8.28
	DSIHE	−18.40*	0.84	0.00	−20.07	−16.74
	RMSHE	3.38*	0.84	0.00	1.71	5.04
	BBCCE	2.58*	0.84	0.00	0.92	4.25

BBCCE	THE	−62.57*	0.84	0.00	−64.24	−60.91
	BBHE	−12.53*	0.84	0.00	−14.20	−10.87
	DSIHE	−20.98*	0.84	0.00	−22.65	−19.32
	RMSHE	0.79	0.84	0.35	−0.87	2.46
	RSIHE	−2.58*	0.84	0.00	−4.25	−0.92

*The mean difference is significant at the 0.05 level.

**Table 6:** Categorization of contrast enhancement methods into homogenous subset using the Duncan test for AMBE.

Method	*N*	Subset for alpha = 0.05				
		1	2	3	4	5
RMSHE	20	14.02				
BBCCE	20	14.82				
RSIHE	20		17.40			
BBHE	20			27.35		
DSIHE	20				35.80	
THE	20					77.39
Sig.		0.35	1.00	1.00	1.00	1.00

Means for groups in homogeneous subsets are displayed.

Harmonic Mean Sample Size = 20.000.

**Table 7:** Categorization of different methods using Fisher's Least Significance Difference (LSD) for FSIM.

(*I*) Method	(*J*) Method	Mean difference (*I*−*J*)	Std. error	*P* value	95% confidence interval	
					Lower bound	Upper bound
THE	BBHE	−0.07*	0.01	0.00	−0.09	−0.05
	DSIHE	−0.05*	0.01	0.00	−0.06	−0.03
	RMSHE	−0.11*	0.01	0.00	−0.12	−0.09
	RSIHE	−0.10*	0.01	0.00	−0.11	−0.08
	BBCCE	−0.19*	0.01	0.00	−0.21	−0.18

BBHE	THE	0.07*	0.01	0.00	0.05	0.09
	DSIHE	0.02*	0.01	0.01	0.00	0.04
	RMSHE	−0.04*	0.01	0.00	−0.05	−0.02
	RSIHE	−0.03*	0.01	0.00	−0.04	−0.01
	BBCCE	−0.12*	0.01	0.00	−0.14	−0.11

DSIHE	THE	0.05*	0.01	0.00	0.03	0.06
	BBHE	−0.02*	0.01	0.01	−0.04	−0.00
	RMSHE	−0.06*	0.01	0.00	−0.08	−0.04
	RSIHE	−0.05*	0.01	0.00	−0.06	−0.03
	BBCCE	−0.15*	0.01	0.00	−0.16	−0.13

RMSHE	THE	0.11*	0.01	0.00	0.09	0.12
	BBHE	0.03*	0.01	0.00	0.02	0.05
	DSIHE	0.06*	0.01	0.00	0.04	0.08
	RSIHE	0.01	0.01	0.12	−0.00	0.03
	BBCCE	−0.09*	0.01	0.00	−0.10	−0.07

RSIHE	THE	0.09*	0.01	0.00	0.08	0.11
	BBHE	0.03*	0.01	0.00	0.01	0.04
	DSIHE	0.05*	0.01	0.00	0.03	0.06
	RMSHE	−0.01	0.01	0.12	−0.03	0.00
	BBCCE	−0.10*	0.01	0.00	−0.12	−0.08

BBCCE	THE	0.19*	0.01	0.00	0.18	0.21
	BBHE	0.12*	0.01	0.00	0.11	0.14
	DSIHE	0.15*	0.01	0.00	0.13	0.16
	RMSHE	0.09*	0.01	0.00	0.07	0.10
	RSIHE	0.10*	0.01	0.00	0.08	0.12

*The mean difference is significant at the 0.05 level.

**Table 8:** Categorization of contrast enhancement methods into homogenous subset using Duncan's test for FSIM.

Method	*N*	Subset for alpha = 0.05				
		1	2	3	4	5
THE	20	0.72				
DSIHE	20		0.77			
BBHE	20			0.79		
RSIHE	20				0.82	
RMSHE	20				0.83	
BBCCE	20					0.92
Sig.		1.00	1.00	1.00	0.12	1.00

Means for groups in homogeneous subsets are displayed.

Harmonic Mean Sample Size = 20.000.

**Table 9:** Ranking of methods in terms of enhancement degree (EME), image quality (FSIM), and mean intensity distortion (AMBE). The methods ranking is computed according to Fisher's Least Significance Difference (LSD) and the Duncan test.

Rank	EME	FSIM	AMBE
1	**BBCCE**	**BBCCE**	RMSHE, **BBCCE**
2	RMSHE	RMSHE, RSIHE	
3	BBHE		RSIHE
4	THE, DSIHE	BBHE	BBHE
5		DSIHE	DSIHE
6	RSIHE	THE	THE
